# mSphere of Influence: Resolution of the Structure of an Influenza Virus Polymerase Is a Game Changer

**DOI:** 10.1128/mSphere.00473-19

**Published:** 2019-08-21

**Authors:** Mathilde Richard

**Affiliations:** a Department of Viroscience, Postgraduate School of Molecular Medicine, Erasmus University Medical Center, Rotterdam, the Netherlands

**Keywords:** influenza, polymerase, structure

## Abstract

Mathilde Richard works in the field of virology, more specifically on the evolution and pathogenesis of influenza viruses. In this mSphere of Influence article, she reflects on how the two articles “Structure of Influenza A Polymerase Bound to the Viral RNA Promoter” by A. Pflug, D. Guilligay, S. Reich, and S. Cusack (Nature 516:355–360, 2014, https://doi.org/10.1038/nature14008) and “Structural Insight into Cap-Snatching and RNA Synthesis by Influenza Polymerase” by S. Reich, D. Guilligay, A. Pflug, H. Malet, I. Berger, et al. (Nature 516:361–366, 2014, https://doi.org/10.1038/nature14009) made an impact on her by providing new grounds to study the influenza virus polymerase and its role in virus biology and evolution.

## COMMENTARY

In 2014, the Cusack research group described the high-resolution structures of the influenza A virus and influenza B virus polymerases in complex with the viral promoter in two articles published back-to-back in Nature (“Structure of Influenza A Polymerase Bound to the Viral RNA Promoter” [1]; “Structural Insight into Cap-Snatching and RNA Synthesis by Influenza Polymerase” [2]). These structures were the first of any negative-strand RNA virus polymerase to be resolved. The genomes of influenza viruses are replicated and transcribed by an RNA-dependent RNA polymerase (RdRp), a heterotrimer consisting of three subunits: PB1, PB2 (polymerase basic proteins 1 and 2, respectively), and PA (polymerase acid protein). The large size and complexity of the multisubunit RdRps often encountered in negative-strand RNA viruses have long hampered their systematic study compared to those of positive-strand RNA viruses. Until the studies by the Cusack research group, only crystals of fragments of the influenza virus RdRp were available, and how the subunits formed a functional protein complex was unclear. The lack of high-resolution structural information of influenza virus RdRps has long prevented advances in understanding fundamental aspects of the virus’ biology and evolution, such as the mechanisms of RNA replication and transcription, mutation generation, and host adaptation. Moreover, the design of novel antiviral strategies targeting the influenza virus RdRp has also been hampered by the lack of structural information.

To overcome technical difficulties of expressing sufficient and pure amounts of influenza virus RdRp necessary for crystallization, the Cusack group produced the polymerase subunits as a self-cleaving polyprotein expressed in insect cells from one single open reading frame (ORF) using the ComplexLINK and MultiBac technologies (3). The genes coding for the different subunits are separated by the cleavage sequence of the NIa protease of the tobacco etch virus (TEV), which is also encoded by the ORF. Upon translation, the TEV protease is the first protein produced and cleaves itself and the other proteins, ensuring the production of all subunits at a stoichiometric ratio. Finally, to bypass difficulties in expressing influenza A virus RdRp from viruses of avian or human origin, Cusack and colleagues crystallized the RdRp from recently discovered bat influenza A viruses. These studies revealed aspects of the influenza virus RdRp in unprecedented detail. It appears that the three subunits are more interconnected than previously assumed. All three subunits take part in most of the key polymerase functions. These studies also are the first to describe the conformation of the active site of the influenza virus RdRp, the sites where the RNA binds, and the putative channels of the template entry/exit, product exit, and nucleotide entry. Moreover, the comparison of the structures of influenza A and B virus RdRps, crystallized in different conformations, revealed the incredible flexibility of the RdRp, in which domains can be rearranged to adapt to carry on multiple roles in viral RNA synthesis.

This work has influenced me in two ways. First, it taught me that perseverance, resilience, and hard work are immense qualities for a researcher. I remember attending a lecture by Cusack in the late 2000s, early in my Ph.D. trajectory. He presented the structures of fragments of the influenza virus polymerase and reported that his group had been working on resolving the structure of the full complex for years but had been unsuccessful because of the technical difficulties of working with such large protein complexes. When this work was published, years after that lecture, I remember being very impressed and thinking, “that’s it, they’ve done it!” Second, both the technological and theoretical advances from this work opened avenues for my own research. The improvement in the biochemistry of the influenza virus RdRp, i.e., the possibility of purifying and producing it in large and pure quantities, allows the development of new assays to study the RdRp in its full context, in the absence or in the presence of the nucleoprotein. Novel techniques that have been successfully used with other purified recombinant RdRps, such as single-molecule assays (4, 5), can now be employed to dissect the kinetics and dynamics of the influenza virus RdRp. Moreover, these new techniques will also be applicable to other negative-strand RNA virus polymerases.

In a broader perspective, these two articles are a game changer for the influenza virus field. The resolution of influenza virus RdRp structures provides an extraordinary basis for future work, both fundamental and applied. In fundamental studies, similar systems have since been used to resolve the structures of RdRps of other influenza viruses: that of the apo (unbound to promoter) influenza C virus RdRp (6), the influenza B virus RdRp in complex with the cRNA promoter (7) and actively transcribing (8), and very recently the apo and promoter-bound influenza D virus RdRp (9). The comparison of structures of influenza virus RdRps in different conformations (free or promoter bound) revealed rearrangements of polymerase domains representing different functional states of the enzyme complex. Additionally, this work will help address some of the most enigmatic aspects of the influenza virus biology, such as the mechanism of replication and transcription of the influenza virus genome, the mechanism of error generation leading to the intrinsic ever-evolving nature of influenza viruses or switch in pathogenicity, and the mechanism of host adaptation mediated by mutations in the influenza virus RdRp. Finally, these studies opened the path to applied research, such as structure-aided drug design targeting the influenza virus RdRp, and helped us to understand mechanisms of action of already-available antivirals and viral strategies to escape them (10).
